# The importance of asking a thorough social history; a case report of aluminum pneumoconiosis

**DOI:** 10.1186/s12890-025-03662-5

**Published:** 2025-04-25

**Authors:** Taylor Caton, Jay Pescatore, Gerard Criner

**Affiliations:** Temple University Hospital, 3401 N. Broad Street, Philadelphia, PA 19140 USA

**Keywords:** Occupational lung disease, Granulomatous lung disease, Pneumoconiosis

## Abstract

Aluminum induced lung disease is associated with pulmonary fibrosis, granulomatous pneumonia, and pulmonary alveolar proteinosis. Recognizing aluminum pneumoconiosis remains important as demand for aluminum production continues to rise. The case presents a 53-year-old woman with incidentally noted lung nodules and hilar lymphadenopathy on CT imaging. Despite being asymptomatic, imaging findings prompted referral to a pulmonologist with CT chest demonstrating bulky mediastinal lymphadenopathy with dominant right paratracheal mass as well as several lung nodules in the bilateral lungs. The patient underwent PET scan revealing hypermetabolic lymph nodes bilaterally in the neck, bones, mediastinum, bilateral hila, retroperitoneal lymph nodes, and spleen. Transbronchial biopsy with nodal aspiration of the mediastium revealed granulomatous inflammation. Treatment was started with prednisone 40 mg daily for presumed sarcoidosis, however she had steroid induced hyperglycemia necessitating multiple hospital admissions. This therapy was then stopped. After eliciting a detailed history, it was noted she worked for 15 years as a powder metal laborer exposed to fine aluminum dust. The case highlights a rare presentation of aluminum pneumoconiosis with diffuse systemic manifestations. Though the exact mechanism of aluminum pathogenesis that produces a sarcoid-like granulomatous reaction remains unclear, metal elements have been implicated in acting as antigens stimulating the immune system. Imaging appearance varies, and CT findings can include alveolitis, small ill-defined centrilobular opacities, lung nodules, and pulmonary fibrosis. Unfortunately, no definitive treatment exists for aluminum pneumoconiosis and limiting ongoing exposure is paramount. Periodic lung function testing and thorax imaging remain staples for monitoring disease progression. Therefore, obtaining a thorough occupational history and awareness of possible manifestations of aluminum pneumoconiosis remains essential as aluminum production and manufacturing demand increases.

## Background

Aluminum exposure has been associated with various pulmonary pathologies. The first documented instances of aluminum lung toxicity were described in the 1930’s [1]. Named Shaver’s disease, aluminum induced pulmonary fibrosis demonstrated an interstitial nonnodular lung fibrosis with upper lobe predominance and peripheral emphysema [2]. Workers exposed to aluminum powder developed symptoms such as dyspnea from anywhere between 1.5 years to over 13 years from exposure [1]. However, multiple other lung pathologies have been reported with aluminum exposure including granulomatous disease, pulmonary fibrosis, and pulmonary alveolar proteinosis [3].

Aluminum production and manufacturing is necessary to a wide variety of industrial sectors, including aerospace, electronics, and automotive. Through 2000, the United States ranked as the world’s largest producer of primary aluminum and remains a major producer of secondary aluminum [4]. Additionally, aluminum demand is expected to increase by 5.1 million metric tons between 2020 and 2030 as North America becomes a larger electric vehicle production location [4]. Consequently, exposure to aluminum powder will increase resulting in potentially higher rates of aluminum pneumoconiosis.

Therefore, further understanding and characterizing the numerous presentations of aluminum pneumoconiosis and taking a thorough workplace exposure history becomes imperative.

## Case presentation

**Fig. 1:**
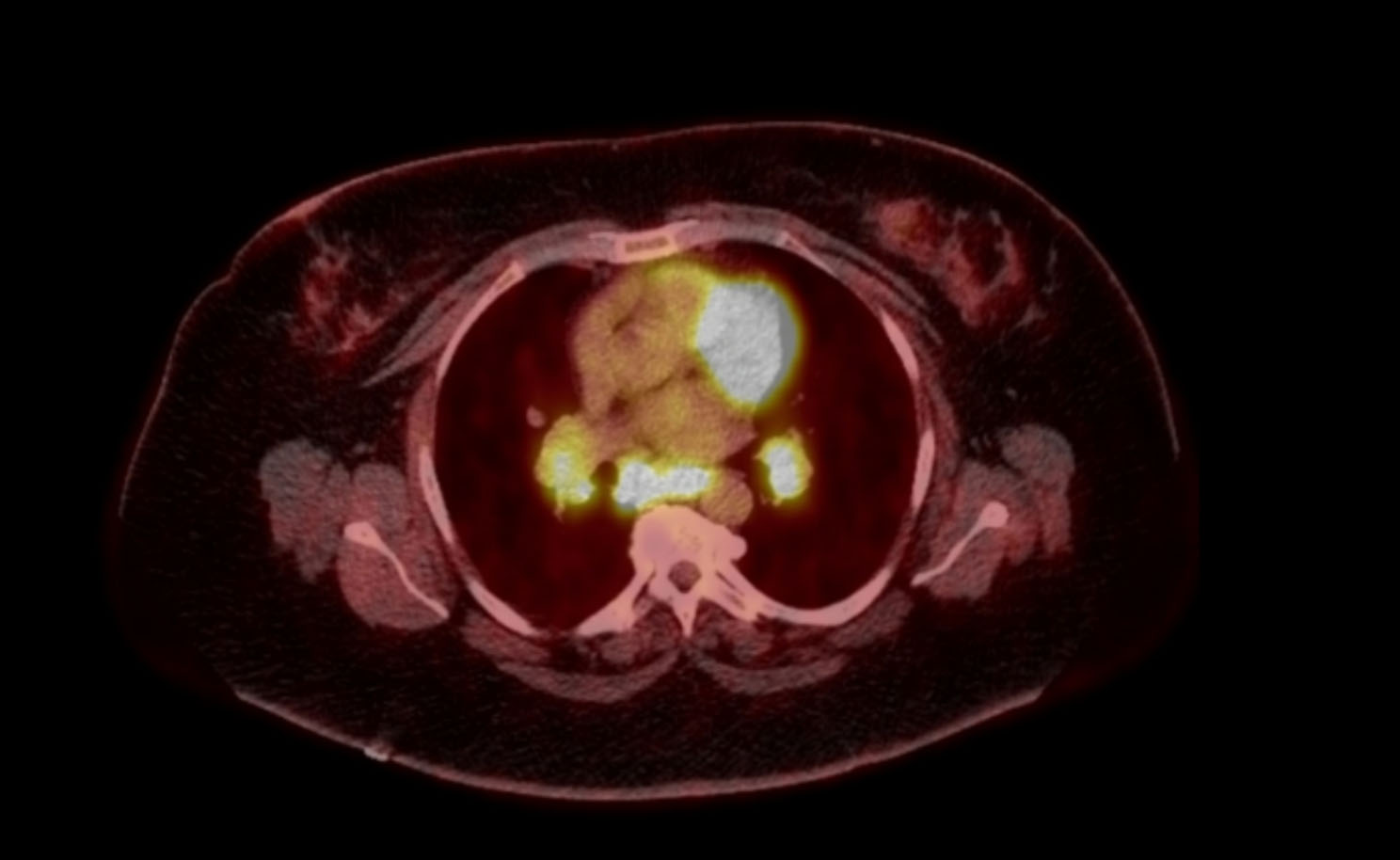
Mediastinal and hilar PET avidity

**Fig. 2:**
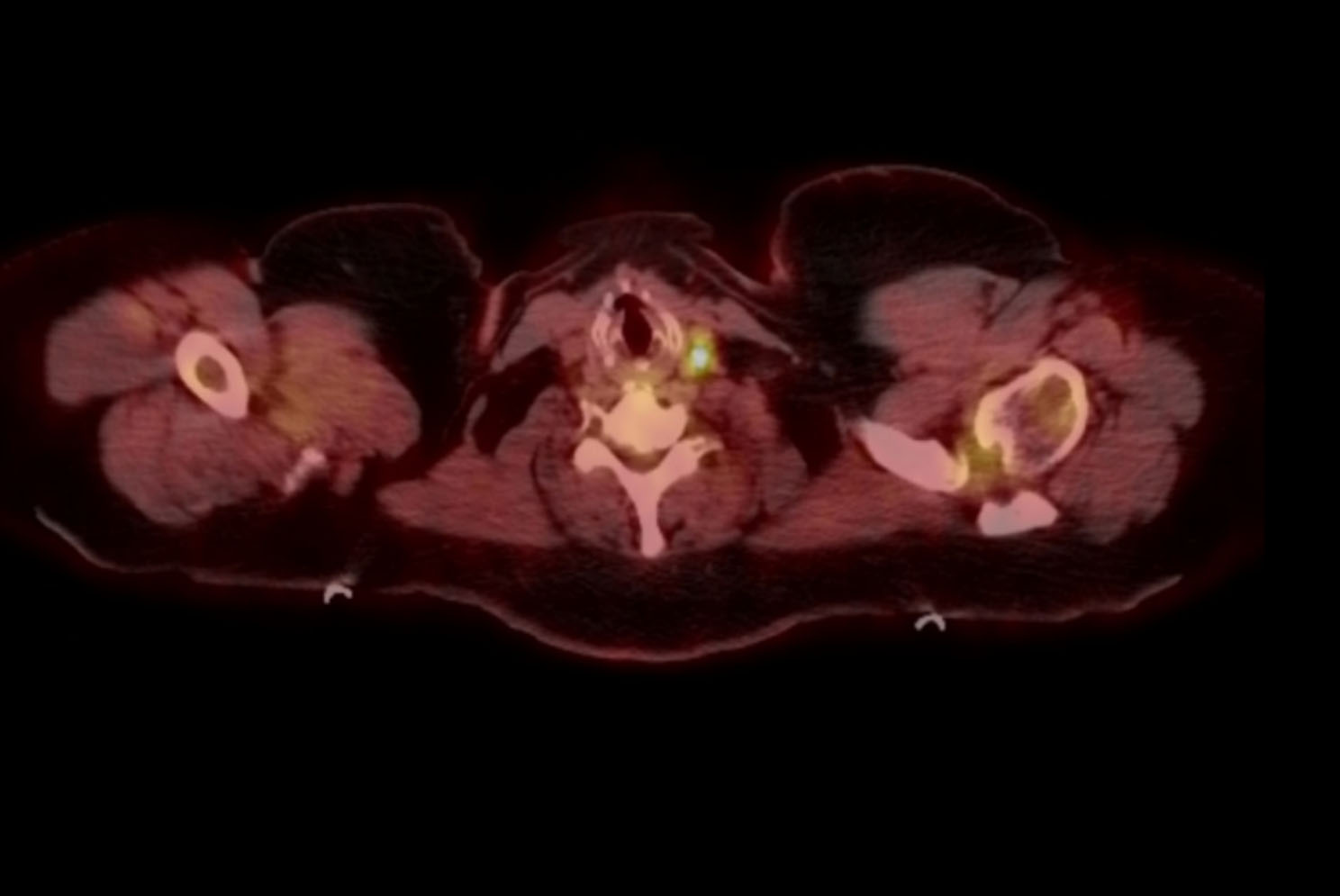
PET scan demonstrating cervical lymph node avidity

A 53-year-old woman presented for a third opinion regarding recent clinical findings. She initially presented to her primary physician for easy bruising over the course of a year without other remarkable physical exam findings. Basic labwork including CBC, BMP, and CXR were normal. She had no documented history or exposures of tuberculosis. However, she was found to have elevated liver enzymes. Viral and autoimmune causes were unremarkable and a CT scan of the abdomen was performed. The scan incidentally noted lung nodules and hilar lymphadenopathy. Initially, the patient denied any respiratory symptomatology. She was referred to a pulmonologist and CT scan of the chest was performed, revealing bulky mediastinal lymphadenopathy with dominant right paratracheal mass measuring 3.6 × 3.1 cm as shown in Fig. 1. Additional small lymph nodes and hilar adenopathy were present. There were lingular and left lower lobe 6 mm nodules, stable 6–8 mm nodules in the right middle lobe, and a 6 mm nodule in the left lower lobe demonstrated on Fig. 2. PET scan revealed hypermetabolic lymph nodes bilaterally in the neck with SUV up to 10.3. Bilateral mediastinal and hilar adenopathy seen previously on CT were redemonstrated with PET avidity. Overall, the PET scan demonstrated evidence of uptake noted in the neck, mediastinum, bilateral hila, rib cage, retroperitoneal lymph nodes, spleen, and pelvic bones. CBC at this time was remarkable for thrombocytopenia to 77 k/uL, WBC 8.77 k/uL and Hgb 13.8 g/dL. To evaluate for metastatic disease, the patient underwent bone marrow biopsy that was negative for granulomas and malignancy. The patient then underwent a transbronchial biopsy with nodal aspiration, revealing granulomatous inflammation. Cultures taken during the procedure were negative for infectious etiologies. Ultimately, patient was presumed to have multisystem sarcoidosis and started prednisone 40 mg daily for a month. However, while on therapy she developed progressive shortness of breath as well as had complications related to steroids including admissions for hyperglycemia, and therapy was therefore stopped.

Regarding past medical history, the patient had an unremarkable family history. She denied living with animals or close contact with livestock. She was a lifetime nonsmoker. Notably, she worked for 15 years as a laborer in the powder metal industry where she was directly exposed to fine aluminum dust. She was employed in various departments at that manufacturer including operating part press, working in the sizing department, and molding. She recalled poor ventilation of the facility, and she noted the plant was often dusty, with aluminum dust falling from the ceiling every time the press was activated. However, OSHA compliance data and aluminum levels at this specific facility were unavailable. After leaving this position, she then took a job inspecting machine parts instead. Differential diagnosis included fungal infections, tuberculosis, autoimmune disease, or malignancy. As mentioned, biopsy results were negative for malignancy, and culture results were negative for infectious etiology. While the patient was initially diagnosed with sarcoidosis, the patient’s symptoms progressed while on steroid therapy making this diagnosis less likely. Therefore, given her social history and prior test results, the patient was then diagnosed with aluminum pneumoconiosis. The patient had already stopped working at that job, limiting further exposure, and her symptomology stabilized after stopping work at the facility in 2023. While there are no diagnostic criteria for aluminum pneumoconiosis due to disease rarity, this diagnosis is suggested by her occupational exposure, biopsy proven granulomatous inflammation, and stability of symptoms after cessation of exposure. She was advised to undergo periodic lung function testing with continued follow up in pulmonology clinic.

### Discussion and conclusions

The case highlights an interesting and rare presentation of aluminum pneumoconiosis with systemic manifestations. Aluminum pathogenesis depends largely on the production of soluble trivalent aluminum ions, typically through exposure to fine aluminum dust or powder [1]. Consequently, the Occupational Health and Safety Administration has limited worker’s exposure to aluminum in dusts to 15 mg/m3 (total dust) and 5 mg/m3 (respirable fraction) of air for an 8-hour workday with 40-hour work week [5]. Though the exact mechanism that produces a sarcoid-like granulomatous reaction remains unclear, metal elements have been implicated to act as antigens to stimulate the immune system even after direct exposure was stopped [3]. Aluminum toxicity is further complicated by variance in its physical and chemical form and may be modified by host factors [1]. Aluminum deposits in the lung may be persistent in triggering an immune response.

Additionally, appearance on imaging can vary for aluminum pneumoconiosis. Often in early stages, CT findings can include alveolitis and small ill-defined centrilobular opacities [6]. Disease progression later appears as upper lobe nodules and pulmonary fibrosis. Interestingly, a cross-sectional study of aluminum powder plant workers who underwent high-resolution computed tomography found that 24.2% of the workers had parenchymal changes [7]. These changes were consistent with aluminosis given demonstration of small, rounded opacities predominantly in the upper lung regions [2]. Occasionally, emphysematous changes and honeycombing may occur ultimately leading to pulmonary fibrosis [6].

More recently, hyperdense nodules have also been associated with aluminum pneumoconiosis. Typically, these nodules are nonspecific and can be associated with a variety of other pathologies including other occupational pneumoconiosis, fungal/mycobacterial infection, sarcoidosis, and cancer [6]. Therefore, distinguishing aluminum pneumoconiosis from other granulomatous lung diseases can be extremely difficult as there is no direct serological, pathologic, or radiographic feature to clinch the diagnosis. Disease diagnosis typically relies on occupational exposure, granulomatous inflammation, and stability of symptoms after cessation of exposure as seen in the case.

In regards to treatment, initially prednisone was provided to the patient for a presumed sarcoidosis. A disease entity known for its steroid responsiveness as it halts the unregulated granulomatous inflammation in response to unidentified/unknown antigens in sarcoidosis. However, in this case steroids did not improve symptoms as well as leading to adverse side effects. Additionally, the granulomatous inflammation seen in aluminumosis is a protective adaptation as it is the immune system walling off the aluminum dust particles. Although a postulation, as there is only limited published literature on aluminum pneumoconiosis, this is an expected inflammatory response to ongoing aluminum inhalation in which patients’ lymphatic system is attempting to clear the dust particles inciting an inflammatory response thus explaining the diffuse avid lymphadenopathy as well. Why the aluminum induced such a significant inflammatory response is unclear. 

There are several limitations to our case report. As this was a third opinion the granular details of the initial work up was limited including the specific types of cultures completed on the biopsies, Tuberculosis testing, as well as blood smear results. However, we do know the results of the culture and bone marrow biopsy were negative. Additionally, due to limitation in laboratory capacity, tissue aluminum levels were not able to be measured.

Overall, the case presents how aluminum exposure can cause systemic manifestations with hypermetabolic nodules. While no specific treatment exists, limiting further exposure is necessary. Periodic lung function testing and thorax imaging remain staples for monitoring disease progression [6]. Therefore, obtaining a thorough occupational history and awareness of possible manifestations of aluminum pneumoconiosis remains paramount especially as aluminum production and manufacturing demand increases.

## Data Availability

All data generated or analyzed during this study are included in this published article.There is no data set for this case report.

## Declarations

Clinical trial number: Not applicable.
